# Genome-wide analysis of acute low salinity tolerance in the eastern oyster *Crassostrea virginica* and potential of genomic selection for trait improvement

**DOI:** 10.1093/g3journal/jkab368

**Published:** 2021-10-25

**Authors:** Alexandra J McCarty, Standish K Allen, Louis V Plough

**Affiliations:** 1 Horn Point Laboratory, University of Maryland Center for Environmental Science, Cambridge, MD 21613, USA; 2 Virginia Institute of Marine Science, Aquaculture Genetics and Breeding Technology Center, Gloucester Point, VA 23062, USA

**Keywords:** *Crassostrea virginica*, oyster aquaculture, salinity tolerance, QTL mapping, genomic selection, BGLR

## Abstract

As the global demand for seafood increases, research into the genetic basis of traits that can increase aquaculture production is critical. The eastern oyster (*Crassostrea virginica*) is an important aquaculture species along the Atlantic and Gulf Coasts of the United States, but increases in heavy rainfall events expose oysters to acute low salinity conditions, which negatively impact production. Low salinity survival is known to be a moderately heritable trait, but the genetic architecture underlying this trait is still poorly understood. In this study, we used ddRAD sequencing to generate genome-wide single-nucleotide polymorphism (SNP) data for four F_2_ families to investigate the genomic regions associated with survival in extreme low salinity (<3). SNP data were also used to assess the feasibility of genomic selection (GS) for improving this trait. Quantitative trait locus (QTL) mapping and combined linkage disequilibrium analysis revealed significant QTL on eastern oyster chromosomes 1 and 7 underlying both survival and day to death in a 36-day experimental challenge. Significant QTL were located in genes related to DNA/RNA function and repair, ion binding and membrane transport, and general response to stress. GS was investigated using Bayesian linear regression models and prediction accuracies ranged from 0.48 to 0.57. Genomic prediction accuracies were largest using the BayesB prior and prediction accuracies did not substantially decrease when SNPs located within the QTL region on Chr1 were removed, suggesting that this trait is controlled by many genes of small effect. Our results suggest that GS will likely be a viable option for improvement of survival in extreme low salinity.

## Introduction

Food insecurity is a global crisis that affects more than a quarter of our population worldwide, but aquaculture provides hope for meeting increasing food demands (FAO *et al.* 2019). Globally, aquaculture has out-produced capture fisheries for a decade (FAO 2018b), and as of 2018, is the fastest growing sector of food production worldwide (FAO 2018a). Marine and coastal aquaculture, specifically, comprised 36% of total aquaculture production in 2016, and nearly 60% of this production came from marine bivalve aquaculture (FAO 2018a). Over the last 20 years, the Chesapeake Bay, located on the eastern seaboard of the United States, has seen substantial increases in eastern oyster (*Crassostrea virginica*) production from aquaculture (Hudson 2018; van Senten *et al.* 2019). As of 2019, there were an estimated 429 total leases comprising 6930 total acres for eastern oyster aquaculture in the Maryland-portion of the Bay (van Senten *et al.* 2019). Oyster harvest increased 115% from 2010 to 2018, and the Maryland shellfish industry was estimated to have an economic impact of over $8 million (van Senten *et al.* 2019). The oyster industry provides a substantial input to the economy of Maryland and also provides valuable employment opportunities in coastal areas where the industry is limited. 

While eastern oyster aquaculture is expanding in the Chesapeake Bay, the highly variable salinity gradient is one of the most prominent environmental factors hindering production for aquaculture operations. Harvest numbers, economic input, and employment associated with the shellfish aquaculture sector in Maryland were substantially lower in 2018 compared to 2017, primarily due to the abnormally low salinity in the Bay resulting from the large inflow of freshwater from heavy rainfall (van Senten *et al.* 2019; NOAA National Centers for Environmental Information 2021). Oyster aquaculture in the upper Bay is periodically faced with the threat of extreme low salinity (<3) resulting from heavy rainfall associated with large storm events. Large mortality events from extreme low salinity (<5) have been observed in estuarine and coastal systems globally (reviewed in Du *et al.* 2021), such as in the Chesapeake Bay (Engle 1946; Andrews *et al.* 1959; Southworth *et al.* 2017), in the Gulf of Mexico (Butler 1949, 1952; Gledhill *et al.* 2020; Du *et al.* 2021), and in northern California (Cheng *et al.* 2015). A lower optimal salinity (∼9–16) has recently been proposed for eastern oyster populations in Louisiana estuaries where freshwater input dominates the hydrodynamics of the system (*e.g.*, La Peyre *et al.* 2016; Rybovich *et al.* 2016; Lowe *et al.* 2017), which is most likely the case for many locations in the northern portion of the Chesapeake Bay. Within the Chesapeake Bay, a “low salinity” oyster line currently exists (salinity ∼6–15; Allen *et al.* 2021), but, growth and survival at low salinity (∼6–15) is arguably different than growth and survival at extreme low salinity (<3) (McCarty *et al.* 2020).

Survival under salinity stress was recently determined to be a heritable trait in the eastern oyster. Survival in high salinity (∼15–23) is a distinct trait from survival in low salinity (∼6–15) (Allen *et al.* 2021), and survival in both low salinity (∼6–15) and in extreme low salinity (<3) have proven to be moderately heritable (salinity ∼6–15 *h*^2^ = 0.34; salinity < 3 *h*^2^ = ∼0.4; McCarty *et al.* 2020). However, genomic (marker-based) analyses of low salinity tolerance in oysters have not been conducted, and knowledge of the genetic architecture of a trait is important when establishing an effective breeding program. Previous genomic investigations of aquaculture traits in the eastern oyster have been focused primarily on resistance to *Perkinsus marinus*, the causative agent of Dermo disease (Yu and Guo 2006). In other aquaculture species, identified quantitative trait loci (QTL) have successfully been incorporated into marker-assisted selection (MAS) programs, for example, for disease resistance in Japanese flounder (Fuji *et al.* 2007), Atlantic salmon (Houston *et al.* 2008; Moen *et al.* 2009, 2015), and Rainbow trout (Liu *et al.* 2018). However, MAS is typically ineffective for most production traits due to their highly polygenic nature, meaning the trait is controlled by many loci of small effect (Zenger *et al.* 2019; Houston *et al.* 2020). On the other hand, genomic selection (GS), or the selection of individuals based on the combined genetic effect of all relevant genome-wide polymorphisms (Meuwissen *et al.* 2001), may be more effective for polygenic traits and produces higher accuracies of selection and higher rates of genetic gain compared to traditional, exclusively performance and family-based, selective breeding programs (Ødegård *et al.* 2014; Zenger *et al.* 2019; Houston *et al.* 2020). Implementation of GS for aquaculture species was once limited to well-studied species such as Atlantic salmon and rainbow trout (Ødegård *et al.* 2014; Vallejo *et al.* 2017; Zenger *et al.* 2019), but recent advances in genomic technology and resources have increased the accessibility of GS for many aquaculture species (Houston *et al.* 2020). The effectiveness of MAS or GS for advancing breeding depends on the genetic architecture of a trait, which is currently unknown for extreme low salinity survival in the eastern oyster.

In this study, we performed QTL mapping and combined linkage disequilibrium (LD) analyses in four F_2_ oyster families exposed to an acute low salinity experimental challenge (<3). Tissue was collected from all individuals, both dead and alive, and genome-wide single-nucleotide polymorphisms (SNPs) generated with ddRADseq (Peterson *et al.* 2012) were used to investigate genomic regions associated with survival and day to death. The potential for using GS to advance breeding of low salinity survival was also investigated by calculating genomic prediction accuracies via cross-validation for several Bayesian linear regression models. This work provides initial insight into the genetic architecture underlying survival in acute low salinity (<3) for the eastern oyster and will help determine whether MAS or GS may provide a better approach for selective breeding of this trait.

## Materials and methods

### F_2_ breeding design

In 2014, 10 F_1_ hybrid families were generated by the Aquaculture Genetics and Breeding Technology Center (ABC) at the Virginia Institute of Marine Science from crosses between individuals from the low salinity and high salinity family breeding lines (Allen *et al.* 2021). In 2015, eight F_2_ families were made by ABC from full-sibling pair-matings within the F_1_ families, and all larvae and seed were reared following standard VIMS protocols (Allen *et al.* 2021). Seed reached ¼ to ½ inch by September and were then transferred to the Horn Point Laboratory (HPL; MD, USA). Once at Horn Point Laboratory, seed were overwintered in the HPL boat basin until March of 2016 when they were put into 3/16-inch vexar mesh bags in a rack and bag setup on the intertidal beach at the Horn Point demonstration farm. Seed were grown in a rack and bag system and checked monthly for biofouling from March to November 2016. From 2016 to 2018, oysters were moved to the HPL boat basin for overwintering from November to March, and then returned to the intertidal demonstration farm from March to November. In March 2018, oysters were transferred to SEAPA^®^ baskets and deployed on an Australian Longline system in the intertidal zone of the demonstration farm at the Horn Point Laboratory until being brought into the laboratory in May 2018 for experimentation. Oysters were ∼3 years old and averaged 92.37 mm ± 0.44 when experimentation began on May 28, 2018.

### Acute low salinity experimental challenge

Oysters from the eight F_2_ families (*N* = 70–140 oysters per family) were randomly divided into equal-sized replicate plastic baskets depending on total number of oysters for each family. Replicate baskets were secured to the bottom of custom-made Taylor floats and submerged in 6-ft diameter tanks (∼1800 L) located indoors at the Horn Point Laboratory in Cambridge, Maryland, USA. Oysters were exposed to acute low salinity (<3) following a very similar experimental design to McCarty *et al.* (2020): a 1-week acclimation period at ambient conditions followed by a 2-day salinity step-down and simultaneous temperature increase. Continuously flowing Choptank River water (salinity ∼7–11) and oxygenated, heated well water (salinity 0) were mixed by hand to maintain salinity 2.3 ± 0.13 and temperature 26.9°C ± 0.07 for 36 days, from May 28 to July 5. A salinity lower than a prior challenge was chosen in hopes of increasing mortality during the experimental timeframe, as only 23% cumulative mortality was observed previously using half-sibling families (experiment 1: April 5–May 7, McCarty *et al.* 2020). Salinity, temperature, and dissolved oxygen were recorded daily with a YSI-85 handheld multimeter (YSI Incorporated, Yellow Springs, OH, USA). Feeding was supplemented daily with Shellfish Diet 1800^®^ (Reed Mariculture, Campbell, CA, USA) at 1.5% total dry tissue biomass and with 3 L of live, cultured phytoplankton from the Horn Point Laboratory Oyster Hatchery. Individual mortality was assessed daily by checking for gaping individuals (McCarty *et al.* 2020), and survival and day of death were recorded for every individual. Adductor muscle was sampled and preserved in 95% ethanol when individuals died, and for all individuals remaining alive at the end of the experiment.

### Library preparation, sequence mapping, and SNP filtering

Four families (11, 43, 22, and 65) were chosen for downstream analysis because they had the largest sample size per family and had the largest range in mortality across the 36-day challenge period (*i.e.*, individual death occurred over many experimental days). A total of 132, 114, 111, and 106 individuals were analyzed for families 11, 43, 22, and 65, respectively. DNA was extracted from a total of 471 tissue samples, 463 samples from the challenge individuals plus the dams and sires of the four F_2_ families, using the E.Z.N.A Tissue Extraction Kit (Omega Bio-tek, Norcoss, GA, USA) following the protocol for preserved animal tissue. Following extraction, DNA concentration was quantified for each sample using a Qubit Fluorometer (Thermo Fisher Scientific Inc., Waltham, MA, USA). Genome-wide SNPs were generated using double digest restriction association DNA (ddRAD) sequencing techniques outlined by Peterson *et al.* (2012). Following digestion with EcoRI and SphI (the “flex-set”), barcoded adapters (1–48) were ligated to each individual separately in a microplate format. Barcoded samples were pooled, and size selection was performed using Agencourt AMPure XP beads (Beckman Coulter Life Sciences, Indianapolis, IN, USA) to select fragments 300–800 bp in length. Size-selected libraries were amplified using the Phusion High-Fidelity PCR Kit (New England BioLabs, Ipswich, MA, USA) and run for 10–12 cycles with a specific indexed primer appropriate for standard Illumina multiplexed paired-end sequencing. Three total libraries were sent to GeneWiz (South Plainfield, NJ, USA) for next-generation single-index sequencing on three Illumina HiSeq 2 × 150 bp sequencing lanes with 15% PhiX spike-in. For each F_2_ family, 5% of the individuals were duplicated to calculate genotype error rate at each locus. GeneWiz demultiplexed libraries based on Illumina indexes, and libraries were further demultiplexed into individual barcoded libraries and renamed using *process_radtags* from the Ddocent pipeline (Puritz *et al.* 2014). All reads from all individuals in the four F_2_ families were grouped together for downstream analysis.

Reads were trimmed and aligned to the eastern oyster reference genome C_virginica-3.0 (GenBank accession GCA_002022765.4; Gómez-Chiarri *et al.* 2015) using the dDocent pipeline with parameters A (match score), B (mismatch score), and O (gap penalty) set to 1, 3, and 5 respectively, which have proven to be more appropriate for marine species (Puritz *et al.* 2014; Dimens *et al.* 2019). After alignment, FreeBayes (version 1.2.0-dirty, Garrison and Marth 2012) was used for SNP discovery and genotype calling, and SNPs were filtered following the dDocent step-wise filtering pipeline for missing data, genotype depth, locus quality score, minor allele frequency (MAF), and genotype call depth (Puritz *et al.* 2014). Individuals with more than 50% missing data were removed, and retained SNPs were present in 90% of individuals, had a minimum read depth of 20 sequences per genotype, a minimum sequence quality score of 30, and a minimum MAF of 0.05. *dDocent_filters* were used to further filter SNPs based on allele balance, quality/depth ratio, mapping quality ratio of reference and alternate alleles, properly paired status, strand representation, and maximum depth using suggested parameters. Polymorphisms were decomposed and indels were removed using v*cfallelicprimitives*. Finally, SNPs were tested for Hardy-Weinberg equilibrium, and SNPs falling below a *P*-value of 0.001 in 25% or more of the population were removed. Parents for one of the families (22) had substantial missing genotype data, so the initial individual missingness filter threshold was relaxed for this family only (individuals with <65% missingness retained). All subsequent filtering steps were identical between family 22 and the other families (*e.g.*, SNP call rate >90%, allelic imbalance, max depth, paired status, and so on). The combined LD analysis (all families combined) was performed on the dataset with initial missingness set at <50% (see below). Genotype error rate (%) was calculated for 21 DNA samples with duplicate RAD library preps (same DNA different barcode) as the cumulative number of mismatches between duplicate genotypes at each SNP divided by the total number of genotypes tested, not including SNPs that had missing data (no genotype call) for either duplicate.

### Linkage map creation, QTL mapping, and combined LD analysis

Linkage maps were created for each of the four F_2_ families independently, and phase information was estimated in OneMap following the Outcrossing Populations tutorial (“OneMap” version 2.1.3; Margarido *et al.* 2007). The package “vcfR” was used to load the raw, filtered SNP file (.vcf) into R for each family before linkage map construction (“vcfR” version 1.9.0; Knaus and Grünwald 2017). For each family, redundant markers and markers with segregation distortion (α < 0.05 after Bonferroni correction) were removed before map building, and only markers present in 90% of the individuals were used. Markers were assigned to linkage groups according to chromosome information from the eastern oyster genome (10 chromosomes, Gómez-Chiarri *et al.* 2015). We thinned each linkage group to 50–100 markers to make mapping easier (*i.e.*, less computation) and because an excessive number of markers are not needed given the architecture of the F_2_ families (*i.e.*, high linkage between markers). Markers were then ordered sequentially according to their location in the genome and phase information was generated using the “map()” function (Margarido *et al.* 2007). A final linkage map was created for each family with linkage groups in correct chromosome order, and the OneMap file outputs were converted to R/qtl format using the OneMap-to-Rqtl-4waycross script (https://github.com/lexymccarty/OneMap-to-Rqtl-4waycross).

QTL mapping was performed in R/qtl for each family independently (version 1.44-9; Broman *et al.* 2003). Individuals with identical genotypes (>90% identical markers) were identified and one individual from each pair was omitted. Markers with identical genotypes (duplicate markers) and markers with segregation distortion [Chi-Square *P* < 0.001] were also removed. Conditional genotype probabilities were calculated (“calc.genoprob”) for each family and a two-part single-QTL model (model=“2part”) was used for phenotype day to death for families 11, 22, and 43 since the phenotype spikes at day 50, representing individuals that survived the low salinity challenge (Broman 2003). In this scenario, we first consider the binary trait where an individual with QTL genotype *g* has probability π_g_ of having the nonzero phenotype (mortality in the low salinity challenge). If the individual has the nonzero value (mortality), the value is assumed to be normally distributed with mean day to death (µ_g_) and standard deviation (σ) (Broman 2003). Therefore, we log-transformed the day to death phenotype to follow a normal distribution. All 2-part QTL models were run with 1000 permutations to determine the 5% significance threshold at the genome-wide level. For family 65, a one-dimensional genome scan was performed with a single-QTL model (“scanone” module) for day to death since all individuals died. Significant QTL were incorporated into a model (“fitqtl” module) to investigate the effect of each QTL on the two traits of interest, survival and day to death, for each family since effect models cannot be fit for 2-part single-QTL models. Finally, “refineqtl” was used to refine the estimated location of QTLs and “fitqtl” was performed on the refined locations to investigate model improvement.

LD analysis was performed on the filtered SNPs from a total of 372 individuals across the four families in TASSEL (version 5.2.57, Bradbury *et al.* 2007). The genotype table was filtered for sites with a minimum MAF of 0.05, maximum frequency of 1, and for sites present in at least 150 individuals (Bradbury *et al.* 2007). Population structure was analyzed using analysis of principle components (PCA; see Results 3.4 Figure 3). Within TASSEL, genotypes from the filtered table were converted to numbers, where the homozygous major genotype is coded as 1, homozygous minor is 0, and heterozygous is 0.5. Missing values were then imputed using Euclidean distance and the 5 nearest neighbors (Bradbury *et al.* 2007). Once all missing values were imputed, a PCA was conducted on the imputed genotype table (Price *et al.* 2006). A kinship matrix using Centered_IBS was calculated from the filtered genotype table to generate pairwise relatedness coefficients for each marker (K matrix) (Bradbury *et al.* 2007).

Combined LD analysis was performed using a mixed linear model (MLM) in TASSEL with the four generated files: the phenotype file filtered for the trait of interest (day to death or survival), the combined filtered genotype table, the first 10 components of the PCA (from TASSEL, explaining 59% of the variation), and the kinship matrix (K) (Bradbury *et al.* 2007; Lu *et al.* 2010):

Phenotypic trait=Marker effect+PCA components+K+residual

Both the PCA and K matrix were used to minimize spurious associations (Lu *et al.* 2010). Two total MLMs were conducted, one for each of the two traits (day to death and survival). Significant thresholds were determined for each model using the Bonferroni correction: α/*N*, where α is the significance level of 0.05 and *N* is the total number of effective tests (determined using “simple” method) to account for any LD between SNPs (Benjamini and Hochberg 1995; Gao *et al.* 2008, 2010). Manhattan plots were created for each trait using the “qqman” package in R (version 0.1.4; Turner 2018). All significant QTLs from the QTL mapping and combined LD analyses were located in the eastern oyster reference genome C_virginica-3.0 (GenBank accession GCA_002022765.4; Gómez-Chiarri *et al.* 2015). Each gene, or gene closest to each significant SNP, was investigated for annotation and function using the NCBI Genome Data Viewer, and corresponding GO terms were queried for each gene/protein sequence (Johnson and Kelly 2020). R version 3.6.1 was used for all necessary analyses (R Core Development Team 2020).

### Genomic prediction and trait correlation

The filtered SNP file used in the combined LD analysis was used to estimate genomic prediction accuracies using Bayesian linear regressions in the statistical package BGLR (version 1.0.8; Pérez and de los Campos, 2014). The genotype file was read into R using the BEDMatrix package (version 2.0.3; Grueneberg and de los Campos 2019) and missing genotypes were imputed using *knncatimpute* in the “scrime” package using the 4 closest neighbors (version 1.3.5; Schwender 2012). Once imputed, genotypes were recoded into BGLR format (AA = 0, Aa = 1, aa = 2) and marker effects were estimated using Bayesian Ridge Regression (BRR) and the BayesB prior for both traits: survival was modeled as a binary trait and day to death was modeled as a censored trait with a minimum value of 0 and a maximum value of 36. The accuracy of marker selection was assessed by randomly splitting individuals into five testing (20%) and training (80%) sets for cross-validation, and phenotypes of the testing individuals were coded as missing in the training set. This process was repeated five times for each trait and each prior. Realized prediction accuracy was calculated as the correlation between the predicted marker values of the testing set and the actual phenotypes divided by the square root of the trait heritability when all phenotypic data is included.

Genomic estimated breeding values (GEBVs) for both traits were estimated in BGLR following the model below:

$$y_{i}=u+{\;Z}_{i}\beta_{i}+e_{i}$$

where *y* is the observed phenotype (either survival or day to death) of the individual, *u* is the average population phenotype, *Z_i_* is the marker-derived matrix of genetic relatedness between individuals (GRM), β_*i*_ is the vector of SNP effects, and *e_i_* is a vector of residual error. Both trait models were fit using a Bayesian Reproducing Kernel Hilbert Spaces Regression (RKHS), but survival was fit with a logit link function because it is a binary trait. Models were assessed by creating five random 20%/80% testing/training validation sets, which was repeated 10 times. Narrow-sense heritability (*h*^2^) was estimated as the additive genetic variance from the GRM over the total phenotypic variance (including both the GRM and residual variance), as follows:

$$h^{2}=\sigma^{2}{}_{a}/\sigma^{2}{}_{p}$$

where σ_*a*_ is the additive genetic variance from the GRM, and σ_*p*_ is the total phenotypic variance, which is the sum of the additive (σ^2^_*a*_) and residual (σ^2^_*e*_) variance. For survival, the residual variance is fixed at 1, so the heritability becomes:

$$h^{2}=\sigma^{2}{}_{a}/\sigma^{2}{}_{a}+1$$

Five independent models were run to estimate heritability for each trait, and the average of the five estimates is reported. Number of iterations, burn in, and thinning parameters were determined by assessing the convergence and autocorrelation for all models using the “coda” package in R (version 0.19-3; Plummer *et al.* 2006; de Villemereuil 2012). All models were run for 2.5 million iterations with a thin of 1000 after a burnin of 500,000 for both traits. For both day to death and survival, BayesB and RKHS models were rerun with the same parameters above, but after removing all SNPs within the significant region on chromosome 1 (21,800,000–32,800,000 base pairs), leaving a total of 27,273 SNPs. Realized accuracies were estimated using the same validation scheme as above. To test the effect of SNP thinning on GS prediction accuracy, we created randomly thinned datasets consisting of 25,000, 20,000, 15,000, 10,000, 5000, 1000, 500, 250, 100, 50, 25, 10, and 2 markers, with three random replicates of each thinned marker dataset. We estimated the realized prediction accuracy for each thinned dataset using a 20%/80% testing/training cross-validation scheme as described above using RKHS and BayesB models. Thinned datasets of 25,000, 20,000, 15,000, and 5000 were omitted for the BayesB models due to computational effort and because the accuracies did not decrease during these intervals. The genetic correlation between the two low salinity challenge traits, survival and day to death, was assessed using a bivariate animal model implemented in ASReml-R using the genotype-derived relationship matrix (Gilmour *et al.* 2015).

## Results

### Survival during the acute low salinity challenge

**Figure 1.**
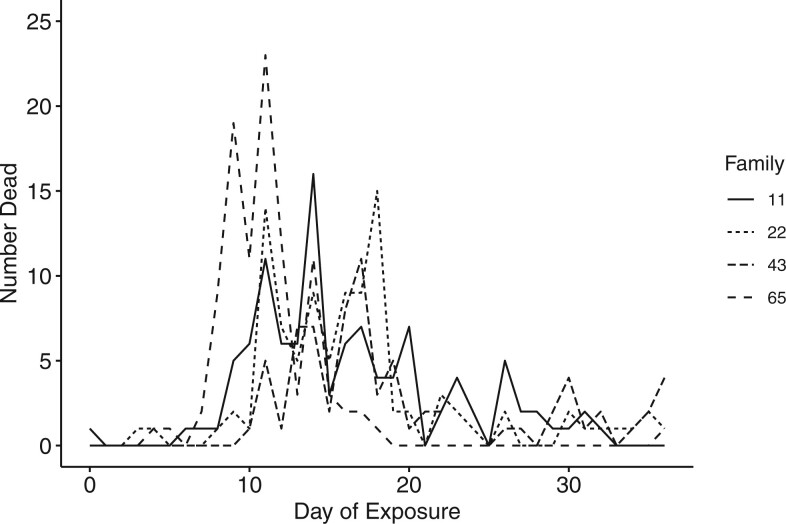
Number dead for each of the four F_2_ families over the 36-day acute low salinity (2.2) challenge. *N* = 132, 111, 114, and 106 for families 11, 22, 43, and 65, respectively.

The acute low salinity challenge (2.2) induced mortality in all four F_2_ families over the 36-day challenge period. 92%, 82%, 90%, and 100% of oysters died from family 22, 11, 43, and 65, respectively. Most of the mortality during the experiment (310 oysters, 80%) occurred from days 9 to 21 of the challenge, with peak mortality occurring on day 11 with 53 total dead oysters (Figure 1). Peak mortality occurred on day 11 for family 22 (14 oysters, 14%) and 65 (23 oysters, 23%), on day 14 for family 11 (16 oysters, 15%), and on day 17 for family 43 (11 oysters, 14%). All families had individuals remaining alive after the challenge except for family 65.

### Sequencing results

All libraries yielded high-quality read data, with >93%, >70%, and >94% of raw reads retained after demultiplexing library 1, 2, and 3, respectively, which resulted in discovery of 4,092,824 SNPs for 489 individuals from the four F_2_ families. The total number of SNPs was significantly reduced after filtering, and the majority of SNPs were removed when filtering for SNPs present in 90% of individuals, a minimum quality score of 30, minor allele count of 3, MAF of 0.05, and a minimum average depth of 20 reads. A total of 28,638 SNPs across 399 individuals remained after applying filters. Mean read depth per site, after accounting for the number of individuals in each group, was 73 and average missingness for each individual was 3.4%. Duplicated samples had an average genotype error rate of 1.97% across all individuals (all families) and within-family error rates ranged from 1.07% (family 22) to 2.74% (family 11).

### Linkage map construction and QTL mapping

**Figure 2.**
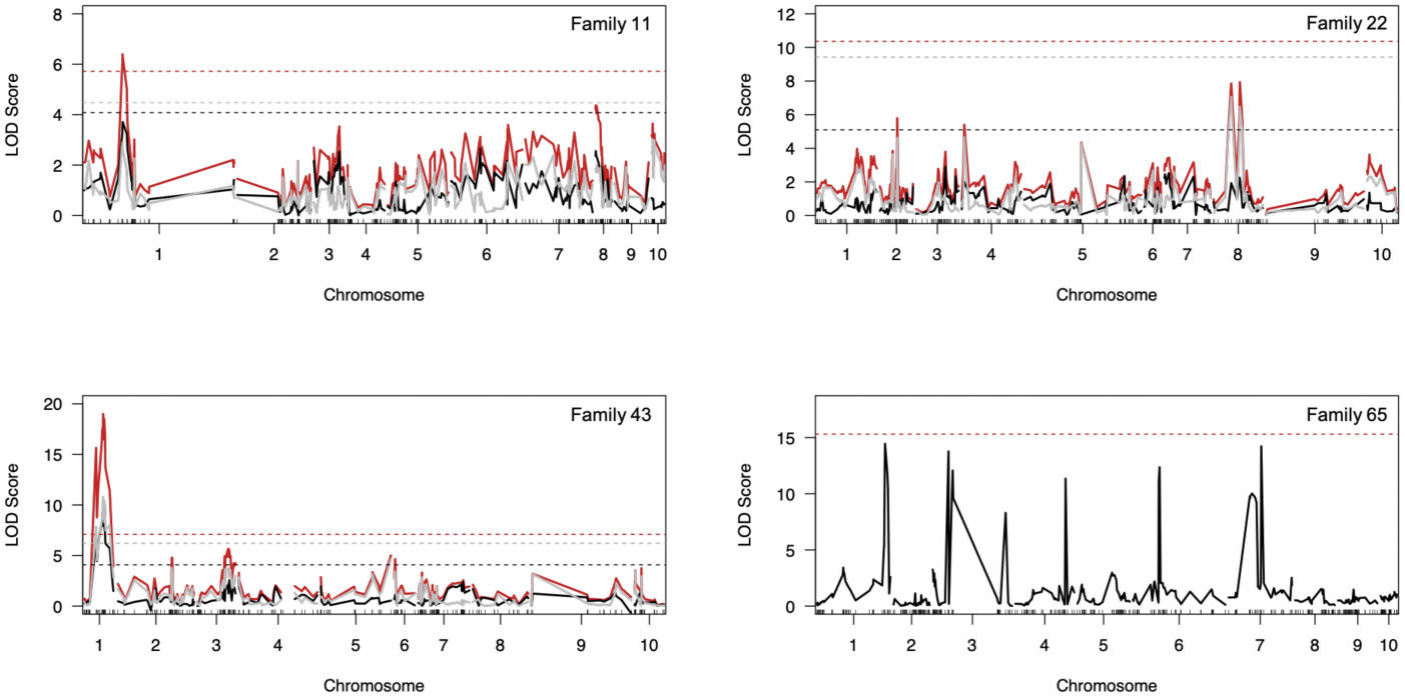
LOD plots for QTL identified from the 2-part model, day to death conditional on survival, for family 11 (top left), 43 (bottom left), and 22 (top right). LOD plot in the bottom right shows the QTL identified from the single-QTL scan for day to death for family 65. For the 2-part models, red lines indicate the QTL associated with mean day to death conditional on the probability of survival (LOD_pµ_), black lines represent QTL associated with the probability of survival (LOD_p_), and gray lines indicate QTL associated with mean day to death (LOD_µ_). Horizontal, dotted lines indicate the 5% significance threshold at the genome-wide level after 1000 permutations for each respective test (by color).

Final linkage maps were created from 123, 100, 91, and 95 individuals using a total of 380, 288, 370, and 400 genotyped markers (after thinning to 50–100 SNPs per chromosome), for family 11, 43, 22, and 65, respectively. A significant QTL on chromosome 1 was identified by the 2-part model (day to death conditional on survival) for family 11 and 43 (Figure 2). All significant markers were located between 21,000,000 and 26,000,000 base pairs on chromosome 1 (Table 1). For Family 11, a significant QTL for the 2-part model (day to death conditional on survival; red line, Figure 2) was located in the uncharacterized LOC111116948 gene on Chr1 in the eastern oyster genome. After incorporating this QTL into a single-QTL model for day to death and after refining the position, the QTL was located in the E3 ubiquitin-protein ligase UBR5-like gene (Table 1) and explained 10.4% of the model variation, but was not above the LOD significance threshold at the genome-wide level. When this QTL was incorporated into the single-QTL model for survival and after position refinement, the QTL was located in the uncharacterized LOC111128605 gene and explained 10.5% of the model variation, but was not above the significance threshold (Table 1). This QTL, before refining, was just below the LOD significance threshold for the probability of surviving from the 2-part model (0.5 below; black line, Figure 2).

**Table 1.** Significant QTL (above the LOD threshold) identified from the 2-part scans incorporated into independent models (“fitqtl”) for both day to death and survival for families 11 and 43. Significant markers were refined for position (“refineqtl”) and then incorporated into each model to get percent variance (% Var) explained and a model significance value (*P*-value). Family 22 and 65 are excluded because there were no peaks above the LOD threshold.

Family	Trait	Chr	Position (bp)	% Var.	*P*-value (χ^2^)	LOD score	Gene
11	Day	1	21,875,299	10.4	0.0136	2.316	E3 ubiquitin-protein ligase UBR5-like
	Survival	1	25,149,524	10.5	0.004	2.915	Uncharacterized LOC111128605*^a^*
43	Day	1	21,924,061	50.55	<0.001	9.32*^b^*	Solute carrier organic anion transporter family member 4A1-like
	Survival	1	25,873,768	32.08	0.001	7.979*^b^*	Nuclear receptor coactivator 2-like

a Gene located closest to the significant QTL.

b Above LOD threshold determined by single-QTL models (“scanone”).

For family 43, the QTL region on chromosome 1 was significant for day to death conditional on survival (red line), the probability of survival (black line), and mean day to death (gray line, Figure 2). This significant QTL was located in the nuclear receptor coactivator 2-like gene in the eastern oyster genome. After incorporation into the single-QTL model for day to death and following refinement, this QTL was above the LOD significance threshold and explained 50.55% of the single-QTL model variation (Table 1). After position refinement, the QTL was located in the solute carrier organic anion transporter family member 4A1-like gene (Table 1). When incorporated into the single-QTL model for survival and after position refinement, the QTL was still located in the nuclear receptor coactivator 2-like gene, explained 32.08% of the model variation, and was above the LOD significance threshold (Table 1). There were no QTL above the significance threshold for families 22 and 65.

### Combined LD analyses

**Figure 3.**
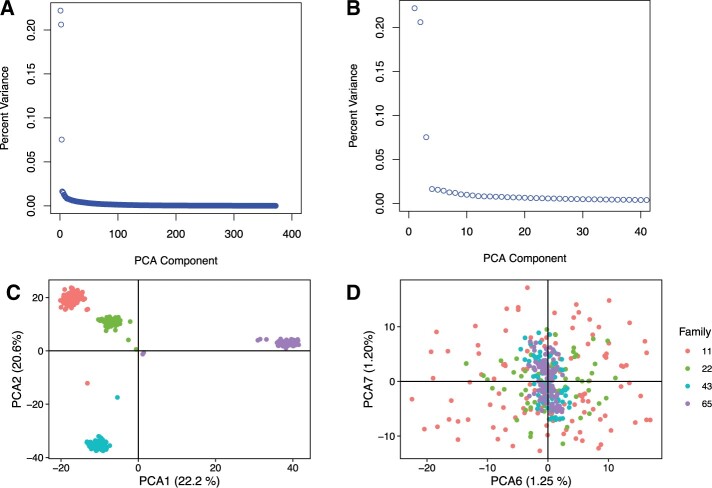
Scree plot showing the percent variance explained by (A) all 372 PCA components and (B) the first 40 components. PCA plots showing (C) population structure when plotting the first two components against each other (*k* = 4), and (D) the lack of structure when components 6 and 7 are plotted.

For the MLMs, the first 10 PCAs (explaining 59% of the variation) were incorporated to account for population structure. When looking at the scree plot of variance explained for each principal component, there was a severe drop after PCA component 3 (Figure 3, A and B). Four distinct populations clustered when plotting PCA components, which represents the four F_2_ families. The population structure completely disappeared when plotting components 6 and 7 (Figure 3, C and D). The scree plot suggests incorporating the first 3 components, while the clustering approach suggests incorporating the first 4. Therefore, we decided to conservatively incorporate the first 10 PCAs, accounting for ∼59% of the variation, into the GLMs to account for population structure in our samples.

**Figure 4.**
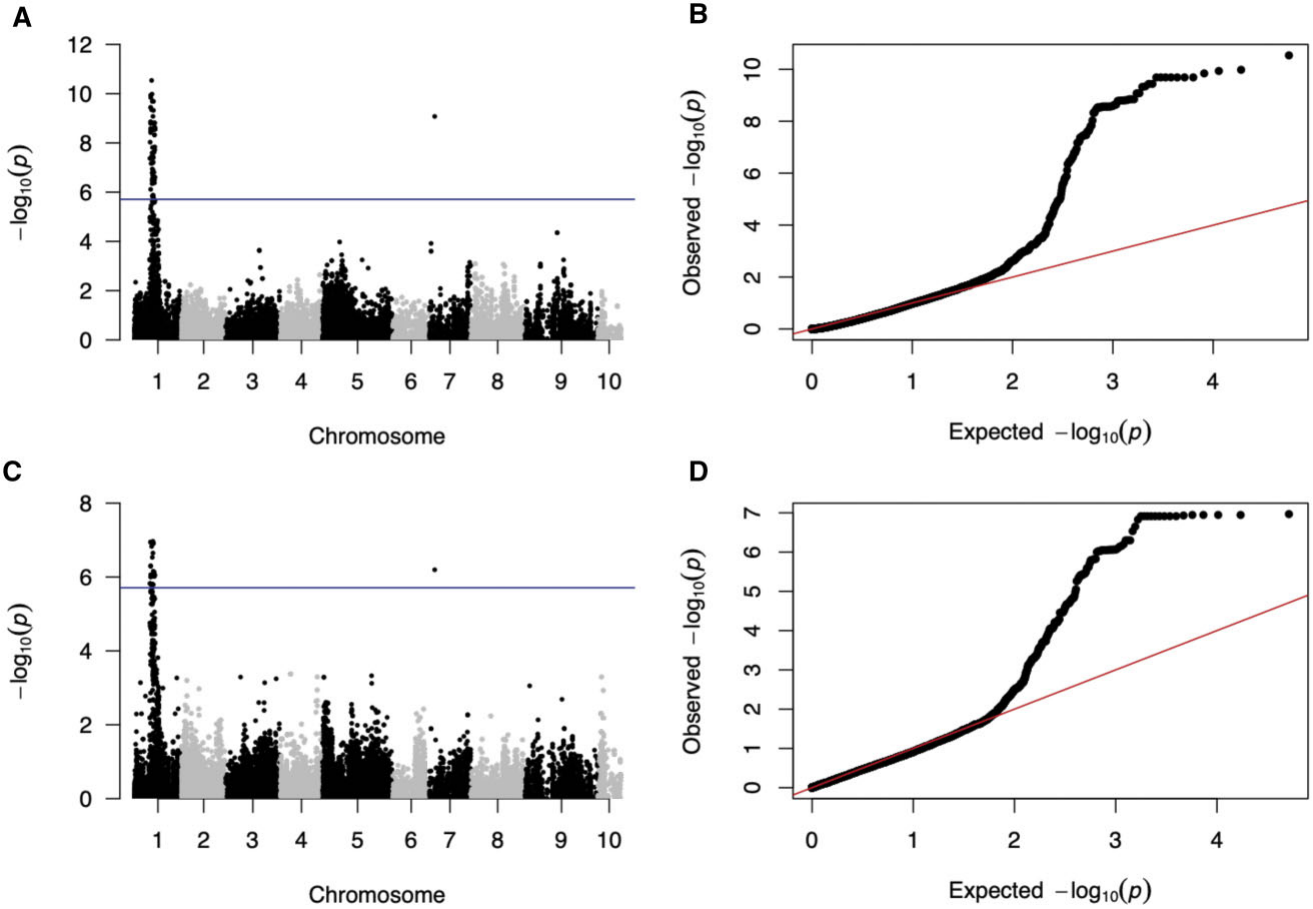
Combined LD analysis of survival (A,B) and day to death (1–36 days, C,D) for the four recombinant families exposed to acute low salinity (2.2) for 36 days. QQ plots (right) and Manhattan plots (left) depicting −log_10_(*p*) values from the combined LD analysis for genome-wide SNPs and survival (A) and day to death (B). Blue horizontal lines in Manhattan plots represent significance threshold after correcting for multiple tests.

The combined LD analysis on a total of 28,502 SNPs identified regions on chromosomes 1 and 7 significantly associated with both survival and day to death. There were a total of 87 and 46 SNPs for survival and day to death, respectively, that were above the significance threshold of 1.95 × 10^−6^ after correcting for the number of effective tests (0.05/25,685 effective tests; Supplementary Table S1; Gao *et al.* 2008, 2010; Yoav and Hochberg 1995). The same 41 SNPs were significant for both day to death and survival, and an additional 5 and 46 SNPs were exclusively significant for day to death and survival, respectively (Supplementary Table S1). Models for both traits revealed a significant peak on chromosome 1 from 21,800,000 to 28,600,000 base pairs, as well as a significant SNP on chromosome 7 at base pair 7,251,580 (significance threshold = −log(1.95 × 10^−6^), Figure 4, A and C).

Comparing between traits (survival and day to death), significant SNPs were located in a total of 16 characterized genes, seven of which were shared between the two traits (Table 2). For the survival MLM, the most significant SNP (chromosome 1, base pair 23,957,309) was not located in a gene, but the next most significant SNP was located in the ATP-dependent 6-phosphofructokinase-like gene with an *R*^2^ value of 0.128 (Supplementary Table S1, Table 2, bold). The most significant SNP (chromosome 1, base pair 25,724,354) from the day to death MLM was also located outside of a gene, but the next most significant SNP was located in the metalloproteinase inhibitor 3-like gene and had an *R*^2^ value of 0.145 (Supplementary Table S1, Table 2, underline). Including all SNPs within the significant QTL peak on chromosome 1 and the single significant SNP on chromosome 7 accounted for 8.97 and 6.51 of the total model variation (*R*^2^) for survival and day to death, respectively (Table 2). When grouping the 16 identified genes by their predicted function, 31% (5 genes) had functions related to DNA/RNA function and repair: coiled-coil domain-containing protein 13-like, E3 ubiquitin-protein ligase UBR5-like, nuclear receptor coactivator 2-like, nucleolar MIF4G domain-containing protein 1-like, and rho GTPase-activating protein 190-like. Another 44% (7 genes) had functions related to ion binding and membrane transport: cadherin-23-like, gamma-aminobutyric acid type B receptor subunit 2-like, metalloproteinase inhibitor 3-like, monocarboxylate transporter 14-like, solute carrier organic anion transporter family member 4A1-like, transient receptor potential cation channel subfamily M member 1-like, and zinc transporter 2-like. The remaining 25% (4 genes) had other predicted functions, such as lipid synthesis and transport and response to oxidative stress: ATP-dependent 6-phosphofructokinase-like, oxidation resistance protein 1-like, extended synaptotagmin-2-like, choline/ethanolaminephosphotransferase 1-like (Table 2).

**Table 2.** Genes with significant SNPs from the combined LD analysis for both survival and day to death in extreme low salinity. Chromosome of annotated gene within the genome is included, along with the significant number of SNPs detected within that gene and their total R^2^. If the gene was present in both analyses, values for survival and day to death are separated by “|”. Gene where most significant SNP was located is bolded and underlined for survival and day to death, respectively. Gene function is indicated with either D (DNA), T (Transport), or O (other).

Trait	Annotated gene	Chr	No. of SNPs	R2	Function
Both	E3 ubiquitin-protein ligase UBR5-like	1	2 | 2	0.171 | 0.234	D
	Metalloproteinase inhibitor 3-like	1	5 | 4	0.583 | 0.584	T
	Monocarboxylate transporter 14-like	1	3 | 2	0.350 | 0.275	T
	Nuclear receptor coactivator 2-like	1	8 | 5	0.856 | 0.715	D
	Nucleolar MIF4G domain-containing protein 1-like	7	1 | 1	0.113 | 0.133	D
	Oxidation resistance protein 1-like	1	3 | 1	0.295 | 0.142	O
	Rho gtpase-activating protein 190-like	1	2 | 2	0.197 | 0.247	D
	Not in a gene	1	25 | 20	2.76 | 2.96	
	Uncharacterized gene	1	18 | 7	1.67 | 0.974	
Survival	**ATP-dependent 6-phosphofructokinase-like**	1	1	0.128	O
	Cadherin-23-like	1	2	0.184	T
	Choline/ethanolaminephosphotransferase 1-like	1	4	0.388	O
	Coiled-coil domain-containing protein 13-like	1	7	0.709	D
	Extended synaptotagmin-2-like	1	2	0.172	O
	Gamma-aminobutyric acid type B receptor subunit 2-like	1	1	0.087	T
	Solute carrier organic anion transporter family member 4A1-like	1	1	0.106	T
	Zinc transporter 2-like	1	2	0.205	T
Day to death	Transient receptor potential cation channel subfamily M member 1-like	1	2	0.247	T
	Total		87 | 46	8.97 | 6.51	

### Genomic prediction, heritability, and trait correlation

**Figure 5.**
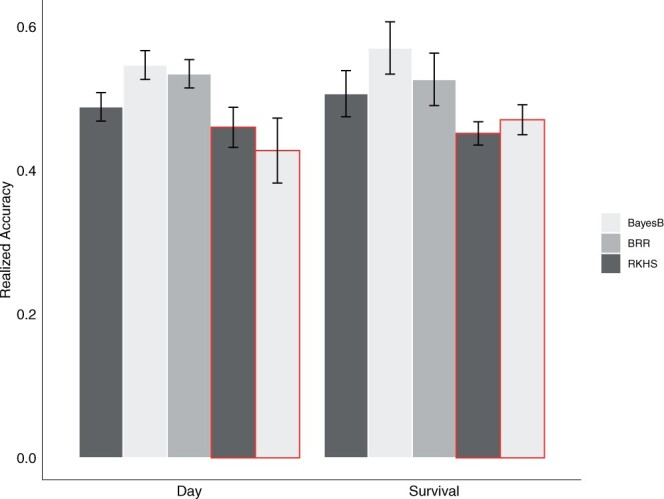
**Realized genomic prediction accuracies for survival and day to death in the extreme low salinity challenge.** Regression models were run for both traits including all SNPs and after removing SNPs in the significant region on chromosome 1 (red outline). Each bar represents the average value of the 50 and 25 separate 20%/80% cross-validation sets for RKHS and marker models (BayesB, BRR), respectively, divided by the square root of the respective estimated heritability value, 0.406 for day to death and 0.539 for survival. Error bars represent standard error of the mean.

Realized prediction accuracies including all SNPs ranged from 0.489 to 0.547 and 0.507 to 0.57 for day to death and survival, respectively (Figure 5). Realized accuracies for both traits were highest for the marker models with the BayesB prior, followed by BRR and RKHS (Table 3). For both traits, removing significant SNPs on chromosome 1 resulted in only a small reduction in accuracy values for both RKHS and BayesB, and the reduction was largest for both traits using BayesB. After removing SNPs on chromosome 1, accuracies for survival decreased by 0.056 and 0.1 for RKHS and BayesB, respectively, and accuracies for day to death decreased by 0.029 and 0.12 for RKHS and BayesB, respectively (Figure 5 and Table 3).

**Table 3.** Realized accuracy estimates (±SEM) for survival and day to death in the acute low salinity challenge. Accuracies were estimated using a 20%/80% testing/training validation set for all regression models (RKHS, BRR, or BayesB) using all SNPs (All) and after removing SNPs in the significant region on chromosome 1 (No Chr1).

Trait	Markers	Model	**Realized accuracy (±SE)** *^a^*
Survival	All	RKHS	0.507 (0.032)
		BRR	0.527 (0.037)
		BayesB	0.571 (0.036)
	No Chr1	RKHS	0.451 (0.016)
		BayesB	0.471 (0.021)
Day to death	All	RKHS	0.489 (0.020)
		BRR	0.535 (0.020)
		BayesB	0.547 (0.020)
	No Chr1	RKHS	0.460 (0.028)
		BayesB	0.428 (0.045)

a Accuracy/√ *h*^2^; *h*^2^ survival = 0.539; *h*^2^ day to death = 0.406.

Realized prediction accuracies decreased when the number of SNP markers used was reduced (thinned) to below 250. For example, realized accuracy dropped to 0.43 and 0.39 for RKHS and BayesB, respectively, when models were run with 100 markers (Supplementary Figure S1).

Narrow-sense heritability estimates were moderate to high for both traits. The heritability estimate for day to death was 0.406 (confidence interval (CI): 0.231–0.595), which is slightly lower than the estimate for survival, 0.539 (CI: 0.326–0.750). The genetic correlation between the two low salinity challenge traits, survival and day to death, was large and significant, 0.867 ± 0.027.

## Discussion

An experimental challenge at extreme low salinity (salinity < 3) was conducted with four F_2_ eastern oyster families to examine the genetic basis of extreme low salinity survival. QTL mapping and combined LD analysis using genome-wide SNPs revealed significant QTL on chromosomes 1 and 7 for both traits, survival and day to death. Genes within, or proximal to, identified QTL had functions related to DNA/RNA function and repair, ion binding and membrane transport, and in the response to stress. Genomic prediction accuracies (0.48–0.57) suggest that GS is a viable option for improving survival in acute low salinity for the eastern oyster, at least based on this dataset. However, future studies with a more appropriate experimental design are necessary. Furthermore, a larger genomic prediction accuracy of the BayesB regression model, along with the lack of substantial decrease in prediction accuracy when removing SNPs within the significant QTL region on chromosome 1, suggest that survival in extreme low salinity may be controlled by many genes of small and potentially unequal effect, as opposed to being controlled by relatively few major-effect QTL.

### QTL and combined LD analyses

QTL mapping and combined LD analysis revealed similar QTL on chromosome 1 related to both survival and day to death. The four significant QTL (Table 1) were located within the significant peak on chromosome 1, from 21,800,000 to 28,600,000 base pairs, detected by the combined LD analysis. For the combined LD analyses, the SNPs located within the significant QTL on chromosome 1 (87 SNPs for survival and 46 SNPs for day to death) explained a total of 8.97% and 6.51% of the total variation for survival and day to death, respectively, with the most significant SNP explaining <0.1% for both traits. For the QTL mapping, a significant QTL explaining a large portion of the total variation (32% for survival and 50% for day to death) was detected for only one of the families (43) after refinement, and the insignificant QTL (after refinement) detected in family 11 explained 10% of the total variation for both traits. The relatively small contribution of our detected (major) QTL from both analyses suggests that many other markers of relatively small effect will likely have a combined large effect on the phenotype. Thus, survival in extreme low salinity (<3) may be controlled by additional genes not identified in this analysis. Aside from one study (Sauvage *et al.* 2010), most QTL studies in bivalve shellfish have examined 2 or fewer families (Zhan *et al.* 2009; Guo *et al.* 2012; Zhong *et al.* 2014; Wang *et al.* 2016; Fang *et al.* 2021).

QTLs of similar magnitude (8%–40% variance explained) identified for multiple traits in salmonids have been proposed for incorporation into MAS programs (Ayllon *et al.* 2015; Barson *et al.* 2015; Gonen *et al.* 2015; Boison *et al.* 2019), but these QTL were validated across multiple populations. In this study, the significant QTL identified on chromosome 1 was detected in only two families, which happen to be the two largest families analyzed, indicating that these families could be driving the detection of the QTL in our combined LD analyses. Thus, the QTL require further validation across other families and populations to determine if they are generally useful and associated with the trait, or if they are specific to the genetic background of the F_2_ families tested. Our sample size was relatively small (<400) and further analyses should be conducted on a larger sample size with individuals from many populations. A sample size larger than 1000 is recommended for higher resolution when detecting QTL (Barría *et al.* 2018; Houston *et al.* 2020), and the possibility of conducting a GWAS of this magnitude is becoming more practical as genomic tools for the eastern oyster continue to be developed (*i.e.*, SNP array; Houston *et al.* 2020; Thongda *et al.* 2018).

QTL mapping and combined LD analyses results suggest that survival and day to death are genetically similar traits. The similarity in the three LOD score curves for the QTL mapping models (2-part: day to death conditional on survival, survival as binary, and day to death with a normal distribution) suggest that similar QTL were identified when analyzing either trait independently. Similarly, the same major QTL region on chromosome 1 was also detected in the combined LD analysis for both traits, and 85 significant SNPs were shared within the same 6.8 million base pair region (21,800,000–28,600,000 base pairs). A high genetic correlation was detected between the two traits (0.867 ± 0.027), and large genetic correlations (0.95) have previously been detected for disease-related survival traits (survival and day to death) in both salmon and trout (Palti *et al.* 2015; Barría *et al.* 2018, 2020; Bassini *et al.* 2019). The finding that survival and day to death are genetically similar suggests that either trait could be used in future assessment of extreme low salinity survival.

Combined LD analysis provided increased resolution and statistical power because all individuals were analyzed together (larger sample size). Combined LD analysis was able to detect not only the significant region on chromosome 1, but also an additional significant region on chromosome 7. Suggestive peaks for the combined LD analysis were present just below the significance threshold on chromosomes 5, 7, 8, and 9, and may contribute to the overall variation in this trait (Figure 4). In contrast, the family-specific QTL analyses did not detect these additional QTL, but there does seem to be a suggestive QTL on chromosome 8 for family 22 (Figure 2). Previous studies have also observed increases in detection ability for combined LD mapping (Xiong and Jin 2000; Lu *et al.* 2010). In our scenario, the combined LD analysis provided the most powerful analysis but is complemented by the independent QTL mapping results.

### Functional analysis of QTL and SNPs

For both survival and day to death, the four major QTL and 133 significant SNPs were located within, or proximal to, a total of 16 annotated genes. These genes have functions belonging to three major categories: DNA/RNA function and repair, ion binding and membrane transport, and the response to stress. Our results build upon previous transcriptomic studies of oysters and highlight potential genes and physiological processes underlying survival in extreme low salinity (<3).

Five of the 16 QTL-associated genes were annotated with functions related to DNA/RNA function and repair. Four of these genes (E3 ubiquitin-protein ligase UBR5-like, nuclear receptor coactivator 2-like, nucleolar MIF4G domain-containing protein 1-like, and rho GTPase-activating protein 190-like) have functions related to RNA binding and gene transcription. Previous work examining the transcriptomic response of eastern oysters to a salinity of 8 and Olympia oysters to a salinity of 5, revealed the strongest enrichment for genes related to DNA replication and transcription (Eierman and Hare 2014; Maynard *et al.* 2018). The enrichment or detection of genes involved in gene transcription at low salinity might reflect the importance, and necessity, of increasing transcription of genes responsible for conformation to stressful low salinities. For example, in eastern oysters, the rho GTPase-activating protein 190-like gene was previously found to be enriched at low salinity (salinity 8) and is considered an important osmoregulatory candidate (Eierman and Hare 2014). Rho proteins are also involved in anti-apoptotic processes (reviewed in Li *et al.* 2015), and infection of Pacific oyster hemocytes with vectors expressing the California sea hare (*Aplysia californica) rho* gene reduced β-adrenoceptor-induced apoptosis (Lacoste *et al.* 2002). Upregulation and expression of many antiapoptotic genes and pathways is a known stress response in oysters (Zhang *et al.* 2016), and rho GTPase-activating protein 190-like may play an important role in preventing apoptosis to maintain internal homeostasis and cell integrity during extreme low salinity exposure.

The majority of genes (7/16) proximal to or underlying QTL were related to membrane transport and ion binding. Oysters are osmoconformers that regulate the concentration of inorganic ions (Na^+^, Ca^2+^, and Mg^2+^) and free amino acids within their cellular fluid to maintain osmotic balance and conform to the salinity of their surrounding environment (Pierce 1971, 1982; Shumway 1977a, 1977b). SNPs significantly associated with variation in low salinity survival were detected in cadherin-23-like and transient receptor potential cation channel subfamily M member 1-like, both of which are transmembrane proteins that play a role in calcium ion binding and cation channel activity (Venkatachalam and Montell 2007; Mège and Ishiyama 2017). Induction of calcium-dependent pathways is a documented response to salinity stress in bivalves (Shumway 1977a; Eierman and Hare 2014; Zhang *et al.* 2016; Gong *et al.* 2021), thereby regulating calcium metabolism, transport, and internal fluid osmolality. In addition, expression of transient receptor proteins are known to be indicative of stress (Venkatachalam and Montell 2007), and are specifically involved in the thermal stress response in both the Pacific and Portuguese oyster (Fu *et al.* 2021). Zinc transporter 2-like gene has functions specifically related to zinc ion binding, and this gene was previously shown to be associated with osmoregulation in the eastern oyster (Eierman and Hare 2014). A significant SNP was also detected in the metalloproteinase inhibitor 3-like gene, which prevents the breakdown of metalloproteins. Metalloenzymes, which are superoxide dismutases with a bonded metal (Cu/Zn or Mn), are part of the defense system against oxidative stress (Rudneva 1999; Park *et al.* 2009). These two genes suggest that zinc ion binding plays an important role in the response to extreme low salinity, and a moderate heritability for zinc ion accumulation in Fujian oysters (Wu *et al.* 2019) could point to a specific mechanism responsible for the observed variation in survival.

The remaining four genes identified from the genome-wide analyses had functions related to oxidative stress and protein regulation. Oxidative stress results from an excess of free radicals in an organism’s cells in response to an environmental stressor (Lushchak 2011; reviewed in Rivera-Ingraham and Lignot, 2017), and expression of antioxidative genes are commonly used to indicate oyster health and stress (Zhang *et al.* 2016). Therefore, the significant SNPs detected in the oxidation resistance protein 1-like gene are not surprising and suggest that extreme low salinity tolerance could be influenced by genetic variation in oxidative response pathways. In addition, maintenance of ion gradients during osmoregulation is one of the most ATP-demanding processes (Hand and Hardewig 1996; Sokolova *et al.* 2012). Many genes involved in protein regulation were previously found to be significantly upregulated in the Pacific oyster after challenge to a low salinity of 8 (Wang *et al.* 2012). The identification of SNPs associated with variation in low salinity tolerance within the ATP-dependent 6-phosphofructokinase-like gene support the notion that glycolysis and energy metabolism are likely important in maintaining cell function during salinity stress.

### Genomic selection and heritability

GS prediction accuracies for all models ranged from 0.48 to 0.57 for both traits, which are slightly lower than ranges reported for production and disease-related traits in other bivalve species. GS prediction accuracies from GBLUP models for growth-related traits ranged from 0.54 to 0.67 and from 0.678 to 0.758 for resistance to Ostreid herpesvirus (OsHV-1-μvar) in the Pacific oyster (Gutierrez *et al.* 2018, 2020). Prediction accuracies reported in studies of other bivalves are relatively similar, e.g. 0.63 - 0.7 for growth related-traits in the Zhikong scallop (Wang *et al.*, 2018) and 0.4 - 0.79 for morphometric and edibility traits in the Portuguese oyster (Vu *et al.* 2021). To our knowledge, there are no reported genomic prediction accuracies for environmental stress-related traits in aquaculture species for comparison, but studies of survival and day to death phenotypes for disease-related traits in finfish species have reported prediction accuracies as low as 0.21 (reviewed in Houston *et al.* 2020). Genomic prediction accuracies are affected by the underlying trait architecture, LD structure, relatedness between training and testing sets, marker density, trait heritability, and sample size (Meuwissen *et al.* 2001; Habier *et al.* 2007; Shengqiang *et al.* 2009; Daetwyler *et al.* 2010; Neves *et al.* 2012; Dou *et al.* 2016; Palaiokostas *et al.* 2019). Therefore, the lower range of the prediction accuracies estimated here may be reflective of the underlying trait architecture.

More likely, the slightly lower range of genomic prediction accuracies may be an artifact of the small sample size (372 individuals) used and the relatedness between training and testing sets (only 4 full-sibling families). The prediction accuracies estimated for low salinity survival in this study are most similar to those reported for growth-related traits in Yesso scallops (GBLUP, BayesB, RRBLUP: 0.3–0.6; Dou *et al.* 2016), where the authors assessed a population size of 349 scallops from 5 full-sibling families. Larger prediction accuracies were found for the Pacific oyster, Zhikong scallop, and Portuguese oyster where more families and larger sample sizes were utilized, *e.g.*, greater than 500 individuals from at least 23 full or half-sibling families (Gutierrez *et al.* 2018, 2020; Wang *et al.* 2018; Vu *et al.* 2021). Caution should be taken when comparing our results to these larger, more comprehensive studies, as our experimental design and F_2_ breeding structure represent fewer families/populations than are typically analyzed in GS studies. Future experimentation with a larger sample size and more populations may increase genomic prediction accuracies, as previous studies have found training population size to have a large effect on prediction accuracies (Ehret *et al.* 2015; Wang *et al.* 2018). Nevertheless, the genomic prediction accuracies estimated here are on par with those in other marine bivalve studies, and larger than some previously reported accuracies for traits in finfish and shrimp species (reviewed in Houston *et al.* 2020).

Substantial thinning (reduction) in marker number (100 markers) was required to observe a noticeable reduction in prediction accuracy (0.07 and 0.18 decrease from the full marker model for RKHS and BayesB models, respectively). This result is likely a consequence of the F_2_ breeding design employed. However, previous studies of marine animals utilizing more families and a more appropriate breeding design (*i.e.*, > 20 half or full-sibling families) have also reported rather subtle decreases in prediction accuracy (∼0.1) when sampling down to hundreds of markers (Gutierrez *et al.* 2018, 2020). Overall, these results suggest that a relatively small number of markers (100 s to a few thousand) may provide adequate genomic prediction accuracies in experimental marine populations utilizing a family-based design. However, future work with larger sample sizes and a more appropriate breeding design is needed before drawing any major conclusions.

For both traits, accuracies were highest for regression models with the BayesB prior, followed by BRR and RKHS regression models. Accuracies differed by 0.064 and 0.058 between BayesB and RKHS models for survival and day to death, respectively, and we suspect that these differences arise from the weighting of the markers. For RKHS models, a traditional animal model replaced by a kernel matrix is executed, which is a matrix of genetic signal (or similarity) between individuals approximated from genetic effects (marker genotypes), as opposed to a traditional GBLUP where the genetic signal is equal to the marker genotypes (Morota and Gianola 2014; Pérez and de los Campos 2014). In RKHS models, one variance is shared and divided between all markers, so each marker is weighted the same and predicted to have the same minimal effect (Meuwissen *et al.* 2001). In BRR, each marker has its own variance, but all are shrunk by the same shrinking parameter (Pérez and de los Campos 2014). Finally, the BayesB prior allows for variable selection, specifically size-of-effect shrinkage, where some markers have a small effect while the rest have minimal to no effect (Meuwissen *et al.* 2001; Habier *et al.* 2011; Morota and Gianola 2014). The slight superiority in model performance by the BayesB prior could reflect the underlying nature of the trait, where survival in extreme low salinity is controlled by a few markers of small effect, such as those on chromosomes 1 and 7, plus additional markers of minimal effect. Similarly, our genomic prediction accuracies decreased only slightly (0.029–0.12) when removing the significant region on chromosome 1, further supporting the notion that survival in extreme low salinity is polygenic in nature, and that regions other than those identified on chromosome 1 contribute to the overall trait variation. It is also worth investigating additional approaches (*i.e.*, GBLUP, BayesA, and BayesC) to ensure we have the model that best reflects the distribution of marker effects on our trait. While a Bayesian model (*e.g.*, BayesB, BBR, BayesA, and BayesC) may fit our trait best, the animal models (GBLUP and RHKS) are easier to implement with faster run times, and differences between GBLUP and Bayesian approaches have proven to be unsubstantial (Zenger *et al.* 2019; Houston *et al.* 2020).

The narrow-sense heritability for both survival (*h*^2^ = 0.539) and day to death (*h*^2^ = 0.406) were very similar to previously reported values using a pedigree-derived relationship matrix between half-sibling families (*h*^2^ ≅ 0.4, McCarty *et al.* 2020). Notably, the heritability estimate for survival was ∼0.133 larger than the heritability estimate for day to death. Previous disease-resistance studies of salmon, red tilapia, and Nile tilapia all reported higher heritability estimates for threshold traits compared to their linear model counterpart (Yáñez *et al.* 2013; Shoemaker *et al.* 2017; Sukhavachana *et al.* 2019), which was suggested to result from a better fit of the threshold animal model for the binary trait (Barría *et al.* 2018). Moreover, higher narrow sense heritability values for disease-resistance traits in both Coho salmon and Nile tilapia corresponded to higher genomic prediction accuracies for these species (Barría *et al.* 2018, 2020), while lower heritability of disease resistance traits in the Portuguese oyster (0.1–0.11) resulted in lower prediction accuracies (0.24–0.3) (Vu *et al.* 2021). The correlation between high heritability values and high genomic prediction accuracies could provide an explanation for the higher genomic prediction accuracies for all survival models in this study.

## Conclusions

Overall, this initial genome-wide analysis indicates that the genetic architecture of survival in low salinity for eastern oysters may be polygenic in nature, with significant QTL located on eastern oyster chromosomes 1 and 7. Moreover, GS appears to be a viable option for improvement of this trait in eastern oysters, which is encouraging as the implementation of GS continues to become more feasible for many aquaculture species. These preliminary results require further validation using larger sample sizes and the inclusion of more families or populations to corroborate detected QTL. Future GWAS experiments will help to elucidate the genomic architecture and the genes underlying low salinity tolerance in oysters, and will ultimately provide more information about the performance of GS for improving this critical trait in oysters.

## Data availability

All raw sequence data are deposited in NCBIs SRA (https://www.ncbi.nlm.nih.gov/sra) under project name (PRJNA756884). Phenotypic information for all individuals, raw and curated markers (SNPs) used in the combined LD analyses and GS models, and genotype files used in Onemap and R/qtl are available in Figshare (https://doi.org/10.6084/m9.figshare.c.5577813).
